# Silk Fibroin-Templated Copper Nanoclusters: Responsive Fluorescent Probes Exhibiting 2,4-Dichlorophenoxyacetic Acid-Enhanced Emission and p-Nitrophenol-Induced Quenching

**DOI:** 10.3390/s26030784

**Published:** 2026-01-24

**Authors:** Neng Qin, Qian Wang, Jingwen Tao, Guijian Guan, Ming-Yong Han

**Affiliations:** 1 Institute of Molecular Plus, Tianjin University, Tianjin 300072, China; qinneng_1@tju.edu.cn (N.Q.); 2024239009@tju.edu.cn (Q.W.); tjw_123_wh@tju.edu.cn (J.T.); han_mingyong@tju.edu.cn (M.-Y.H.); 2 State Key Laboratory of Advanced Papermaking and Paper-Based Materials, South China University of Technology, Guangzhou 510640, China

**Keywords:** silk fibroin, nanoclusters, fluorescence, sensor, pollutant

## Abstract

In this work, highly water-soluble silk fibroin (SF) is first prepared by recrystallizing degummed silkworm cocoon fibers in concentrated CaCl_2_ solution (replacing the conventional Ajisawa’s reagent), and then used as both stabilizing and reducing agents to synthesize copper nanoclusters (Cu@SF NCs) at pH = 11. Due to the existence of unreacted Cu^2+^ ions, the resulting SF-templated Cu NCs form slight aggregates, yielding a purple-colored solution with blue fluorescence. Interestingly, upon adding the pesticide 2,4-dichlorophenoxyacetic acid (2,4-D), the Cu NCs aggregates disassemble and the fluorescence is significantly enhanced, creating a “fluorescence-on” sensor for 2,4-D with a detection limit of 0.65 μM. In contrast, the pollutant p-nitrophenol (p-NP) quenches the fluorescence of Cu NCs via a fluorescence resonance energy transfer mechanism (with a detection limit as low as 1.35 nM), which is attributed to the large overlap between absorption spectrum of p-NP and excitation spectrum of Cu NCs. Other tested analytes (i.e., pyrifenox, carbofuran and melamine) produce negligible fluorescence changes. The distinct sensing mechanisms are elucidated with experimental evidence and density functional theory (DFT) calculations. The evolutions of fluorescence as a function of incubation time and analyte concentration are systematically investigated, demonstrating a versatile platform for sensitive and selective detection of target analytes. These findings provide an effective strategy for optimizing the optical properties of metal nanoclusters and improving their performance in environmental applications.

## 1. Introduction

Fluorescent materials have been extensively developed in recent decades for applications in optical displays, chemical sensing, and biological imaging, owing to their unique light-emitting properties across various wavelengths [1]. In particular, semiconductor quantum dots (QDs) have been synthesized by wet-chemical approaches to replace organic fluorescent dyes, offering large Stokes shifts and improved photostability that mitigate fluorescence bleaching over time. QDs feature component-, size-, and structure-dependent excitation/emission properties and relatively narrow emission bands [2,3,4]. In recent years, few-atom metal nanoclusters (NCs) have gained considerable attention as ideal alternatives to organic dyes and QDs. Metal NCs not only exhibit excellent photostability and large Stokes shift, but also offer lower toxicity and much smaller size, affording better biocompatibility for biological applications [5,6,7].

Benefiting from chemical stability and facile preparation, significant progress has been made in the synthesis of gold and silver NCs, achieving tunable surface features, strong fluorescence, and improved performance via precise size control and structural tailoring [8]. In contrast, research on copper NCs remains in an early stage, despite copper’s abundance, low cost, and widespread industrial use [9]. Synthesizing Cu NCs with well-controlled size is challenging, and the as-prepared Cu NCs tend to aggregate (resulting in poor dispersibility) and oxidize upon exposure to air [10,11]. Therefore, new strategies to obtain stable, highly fluorescent Cu NCs and to broaden their application fields are still highly desirable, given copper’s abundant availability and economic advantages.

Cu NCs are typically synthesized via a bottom-up method, where Cu^2+^ ions are reduced to Cu atoms and subsequently accumulate into clusters [12,13,14,15]. To prevent oxidation and aggregation of Cu NCs, appropriate stabilizing ligands are indispensable during the reaction or via post-synthesis surface treatments [16,17,18]. Thiol-containing small molecules such as glutathione (GSH) and L-cysteine have been widely used as capping ligands and reducers for Cu NC synthesis, since thiols can both reduce metal ions and bind strongly to metal surfaces [9,16,19]. Typically, mixing GSH with Cu^2+^ ions at pH 6 and applying ultrasonic treatment for 15 min produced GSH-capped Cu NCs with red fluorescence [19]. When the reaction pH is raised to 10.0 and the mixture is incubated at 40 °C for 14 h, GSH-protected Cu NCs emitting blue fluorescence were obtained [20]. Similarly, L-cysteine can act as both a capping and reducing agent (in the presence of NaOH) to synthesize Cu NCs [21].

In addition to small thiols, larger molecules, such as polymers [22], oligonucleotides [23], peptides [24], and proteins [25], have also been employed as scaffolds or templates to prepare stable Cu NCs. These functional ligands have enabled the synthesis of Cu NCs with tunable emission wavelengths and high fluorescence, offering potential for large-scale applications [26]. Among these approaches, protein-templated synthesis is especially attractive for improving the water solubility and biocompatibility of Cu NCs, as proteins could serve as environmentally benign reducing and stabilizing molecules [10]. For instance, bovine serum albumin (BSA) has been used (with NaOH) to reduce and stabilize Cu NCs, yielding blue emission under excitation at 325 nm with a quantum yield of 0.15% [25]. Additionally, transferrin-templated Cu NCs have also been prepared by reducing Cu^2+^ with ascorbic acid under basic condition [27]. More recently, trypsin-stabilized Cu NCs with strong blue emission were synthesized at room temperature using hydrazine hydrate as a mild reductant [28]. With the rapid development of this field, an increasing variety of proteins have been explored to fabricate Cu NCs.

It is well known that ligands can significantly influence the optical properties of Cu NCs, including fluorescence intensity and emission wavelength [29]. Moreover, surface ligands affect the sensitivity and selectivity of Cu NCs-based sensors, underscoring the importance of selecting appropriate capping molecules to create high-performance sensing materials [30]. For example, BSA, being a globular protein, fully encapsulates Cu NCs to stabilize them; however, this complete coverage can reduce the NCs’ sensitivity to target molecules. In contrast, silk fibroin (SF), a fibrous protein, can provide a more exposed surface on Cu NCs, potentially affording better interaction with analytes and thus improving sensor performance. In this work, we used SF as both the template and the reducing agent to synthesize Cu NCs that are initially slightly aggregated due to the existence of unreacted Cu^2+^ ions and exhibit blue fluorescence. Owing to the strong binding affinity of deprotonated 2,4-dichlorophenoxyacetic acid (2,4-D^−^) with Cu^2+^ ions, adding 2,4-D causes the aggregated Cu NCs to re-disperse, resulting in a greatly enhanced fluorescence (“turn-on”) response for 2,4-D. In contrast, another pollutant, p-nitrophenol (p-NP) induces pronounced fluorescence quenching of the Cu NCs via a fluorescence resonance energy transfer mechanism. Other tested molecules did not bind effectively to the Cu^2+^ ions and thus produced no significant fluorescence change. These distinct and selective responses form the basis of a novel sensing platform for rapid detection and discrimination of specific analytes.

## 2. Materials and Methods

### 2.1. Chemicals

Protease (from *Bacillus* sp.) and the pollutant standards 2,4-D, p-NP, pyrifenox (Py), carbofuran (CBF), melamine (Me), 2-methyl-4-chlorophenoxyacetic acid (MCPA) and dicamba were purchased from Sigma-Aldrich. *Bombyx mori* silkworm cocoons were obtained from Carolina Biological Supply Co. Copper(II) chloride dihydrate (CuCl_2_•2H_2_O), calcium chloride dihydrate (CaCl_2_•2H_2_O), sodium bicarbonate (NaHCO_3_), hydrochloric acid (HCl, 35%), sodium hydroxide (NaOH), and ethanol were purchased from Tianjin JiangTian Chemical Engineering Co., Ltd. (Tianjin, China). Double-distilled water was used for all the experiments.

### 2.2. Preparation of SF Solution

SF solution was prepared by degumming and dissolving silkworm cocoons. Briefly, the cocoons were cut into small pieces and boiled at 60 °C for 2 h in an aqueous solution of sodium bicarbonate (1 mg/mL) and protease (10 mg/mL) to remove sericin. The degummed silk was then dissolved in a 50% (*w*/*w*) aqueous CaCl_2_ solution at 135 °C for 4 h. The resulting SF solution was dialyzed against distilled water for 48 h (with six water changes) and then centrifuged at 9000 rpm (rcf = 9146× *g*) for 20 min at room temperature to remove any insoluble matter. This process yielded a clear, pale-yellow SF solution (i.e., supernatant). The SF concentration was determined to be ~12.5 mg/mL by drying a measured volume of the solution and weighing the residual protein.

### 2.3. SF-Induced Synthesis of Copper Nanoclusters (Cu@SF NCs)

Cu@SF nanoclusters were synthesized using SF as the template and reductant. In a typical experiment, 1 mL of 10 mM CuCl_2_ aqueous solution was added to 4 mL of SF aqueous solution (12.5 mg/mL) under vigorous stirring at room temperature. After stirring for 10 min, 50 μL of 1 M NaOH solution was added to raise the pH. The mixture was then incubated at 40 °C for 4 h. As a result, a slight purple-colored Cu@SF solution was obtained, which exhibited a blue fluorescence when excited at 324 nm.

### 2.4. Fluorescence Response of Cu@SF NCs to Pollutant Addition

Stock solutions of different pollutants with various concentration were prepared by dissolution and dilution in ethanol. For fluorescence sensing measurements, 450 mL of the Cu NCs solution was placed in a quartz fluorescence cuvette and its initial fluorescence intensity (F_0_) at 400 nm was recorded. Then, 50 μL of a pollutant stock solution was added, and the mixture was incubated for 2 h before measuring the final fluorescence intensity (F). The fluorescence response was quantified as the ratio Q = F/F_0_, where Q > 1 indicates fluorescence enhancement and Q < 1 indicates quenching. The time-dependent fluorescence evolution after analyte addition, as well as the fluorescence response at varying analyte concentrations, were also recorded to evaluate the sensor’s response kinetics and sensitivity.

### 2.5. Characterization

UV–visible absorption spectra were recorded on a Shimadzu UV-3150 spectrophotometer. Photoluminescence spectra were collected using a Shimadzu RF-5301PC spectrofluorometer. Optical photographs of solutions were taken under ambient light with a Canon EOS 350D digital camera, and fluorescence photographs were taken under 365 nm UV illumination using the same camera. X-ray photoelectron spectroscopy (XPS) was performed on a Thermo Fisher ESCALAB Xi instrument (WSx, UK) to analyze the copper oxidation states. The molecular weight distribution of SF was analyzed by SDS-PAGE (sodium dodecyl sulfate polyacrylamide gel electrophoresis). The gel was run at 250 mA with a pre-stained protein marker (3–200 kDa, Thermo Fisher, MA, USA) and stained after electrophoresis to visualize the protein bands.

### 2.6. Calculation of Binding Energies

First-principles calculations were carried out using density functional theory (DFT) with the Vienna Ab Initio Simulation Package (VASP). The projector augmented wave (PAW) method and the PW91 exchange-correlation functional were employed with an energy cutoff of 400 eV. To model interactions, we computed the energies of isolated species (e.g., a deprotonated 2,4-D anion) and combined complexes (e.g., 2,4-D^−^ adsorbed on a Cu^+^ ion) after full geometry optimizations. A vacuum spacing of 15 Å was applied in all simulations to prevent interactions between periodic images, and a single k-point was used for Brillouin zone sampling. Structural relaxation was performed until the forces on all atoms were less than 0.005 eV/Å. For 2,4-D^−^, three possible binding configurations on a Cu^+^ ion were considered, and the lowest-energy optimized structure was used for binding energy calculations. The binding energy (E_bind_) of 2,4-D^−^ on Cu^+^ was defined as the sum of the energies of the separate Cu^+^ cation and 2,4-D^−^ anion minus the total energy of the Cu^+^–(2,4-D^−^) complex.

## 3. Results and Discussion

### 3.1. Preparation of Highly Soluble Silk Fibroin

*Bombyx mori* silk fiber consists of a core of silk fibroin coated with a layer of silk sericin (SS), and it has long been used in biomaterials and textiles [30,31]. Silk is prized for its remarkable mechanical strength, biocompatibility, controlled biodegradability, and high optical transparency (90–95% in thin films) [32,33,34]. Raw silk fibers contain two classes of proteins: SF (>75% by weight) and SS (<25%, a glue-like protein that binds fibroin fibers) [35,36,37]. The SF protein is composed largely of glycine (Gly) (~43%), alanine (Ala) (~30%), and serine (Ser) (~12%) residues, and consists of a heavy chain (~350 kDa) and a light chain (~25 kDa) linked by a single disulfide bond [36,38].

**Figure 1:**
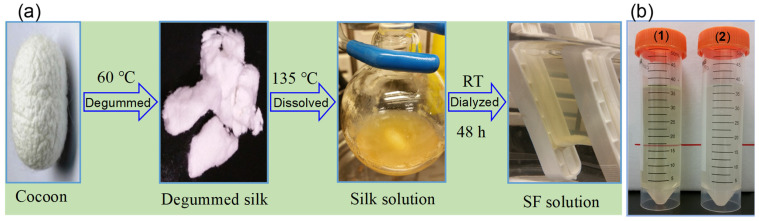
Preparation of regenerated silk fibroin (SF) from silkworm cocoon. (**a**) Optical images illustrating the SF extraction process: successive degumming of the cocoon in a protease/NaHCO_3_ solution at 60 °C, dissolution of the degummed silk in 50% aqueous CaCl_2_ solution at 135 °C, and purification by dialysis in water at room temperature (RT). (**b**) Optical comparison of SF solutions prepared using (1) 50% CaCl_2_ solution and (2) Ajisawa’s reagent (CaCl_2_/H_2_O/C_2_H_5_OH =1:8:2, molar ratio) after storage at room temperature for one month.

A critical step in obtaining pure SF is the degumming process, which removes the sericin coating. Residual sericin can induce inflammatory reactions [39] and makes silk fibers difficult to dissolve [40]. Moreover, sericin remaining on SF masks active binding sites and disrupts SF’s coordination with metal ions, hindering the formation and performance of SF-templated nanomaterials. Therefore, effective degumming is essential to obtain high-purity SF for functional applications. Traditional methods use Ajisawa’s reagent (a CaCl_2_/ethanol/water ternary solution) to dissolve SF. However, due to the very high molecular weight of SF and its extensive β-sheet structure, fibroin regenerated with Ajisawa’s reagent tends to be hydrophobic and quickly forms gels (within about one week) [41]. In this study, we adopted a different approach using a 50% (*w*/*w*) CaCl_2_ solution to extract regenerated SF (Figure 1a). Silk cocoons were first degummed in a NaHCO_3_/protease solution to remove sericin, and the resulting fibroin fibers were then dissolved in hot 50% CaCl_2_. After dialysis and centrifugation, this method yielded a stable, clear SF solution.

The molecular weight distribution of the regenerated SF was relatively broad, ranging approximately from 3 kDa to 49 kDa (Figure S1). Under identical conditions, the SF solution obtained via the 50% CaCl_2_ method showed significantly greater stability and solubility than that prepared with Ajisawa’s reagent, as evidenced by the lack of gelation over time (Figure 1b) [42]. Spectroscopic characterization of the purified SF (Figure S2) revealed a strong UV absorption at 273 nm and a weak intrinsic fluorescence emission at 308 nm when excited at 260 nm. This faint fluorescence is attributed to trace organic compounds naturally present in silk (e.g., flavonoid and carotenoid pigments) [43,44,45].

### 3.2. Synthesis and Optical Characterization of SF-Templated Cu NCs

**Figure 2:**
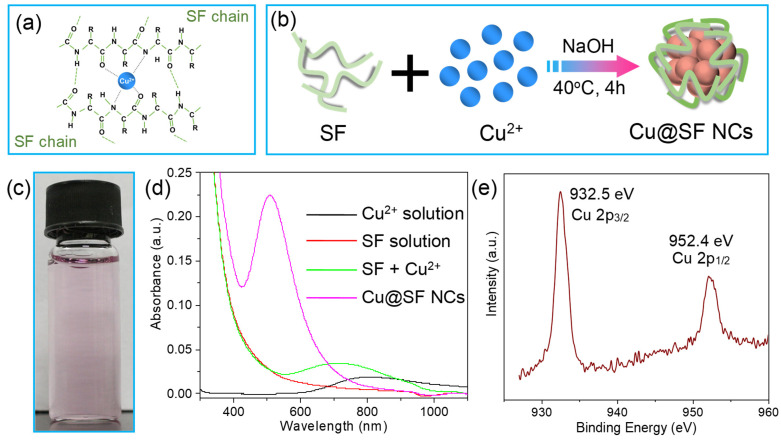
SF-induced synthesis of Cu@SF NCs and their characterization. (**a**) Proposed binding configuration of Cu^2+^ ion with SF chains. (**b**) Schematic illustration of the one-pot synthesis of Cu@SF NCs using SF as both reducing agent and stabilizer. (**c**) Optical image of the as-synthesized Cu@SF NC solution under visible light. (**d**) UV-vis absorption spectra of the Cu^2+^ precursor solution (black trace, peak at 806 nm), SF solution (red trace), the Cu^2+^ + SF mixture before reduction (green trace, peak at 715 nm), and the final Cu@SF NC solution after reduction (pink trace, new peak at 508 nm). (**e**) XPS spectrum of Cu@SF NCs, showing Cu 2p_3/2_ (932.5 eV) and Cu 2p_1/2_ (952.4 eV) peaks with the absence of any Cu^2+^ signal, confirming that Cu^2+^ has been reduced to Cu^0^/Cu^+^.

During the Cu NC synthesis, SF acts as both the reducing agent and the stabilizing template. The oxygen and nitrogen atoms on SF amino acid residues can coordinate with Cu^2+^ ions [46], facilitating effective binding of Cu^2+^ to the protein chains (Figure 2a). Upon adjusting the solution to alkaline pH, certain residues in SF (such as Tyr and Ser) become deprotonated and are able to reduce Cu^2+^ to Cu^0^/Cu^+^ [47]. Following this one-pot, green synthesis approach, we obtained water-soluble, blue-luminescent Cu NCs templated by SF (Figure 2b). The final product is a pale purple solution of Cu@SF NCs (Figure 2c). For comparison, the CuCl_2_ starting solution is light blue, the SF solution is colorless, and a simple mixture of Cu^2+^ and SF appears light blue; only after NaOH addition and 4 h of reaction at 40 °C does the characteristic light purple color of Cu NCs develop (Figure S3).

As shown in Figure 2d, The UV–vis spectrum of the Cu@SF NC solution exhibits a new absorption band centered at ~508 nm that is not present in the Cu^2+^/SF mixture prior to NaOH addition. This indicates the formation of slightly aggregated copper nanoclusters with distinct optical properties. XPS analysis further confirmed the reduction of Cu^2+^. In the Cu@SF NCs, the Cu 2p_3_/_2_ and 2p_1_/_2_ peaks appear at 932.5 eV and 952.4 eV, respectively, corresponding to Cu^0^/Cu^+^ species (Figure 2e) [48]. Together, these results verify the successful synthesis of zerovalent/monovalent Cu nanoclusters stabilized by SF.

**Figure 3:**
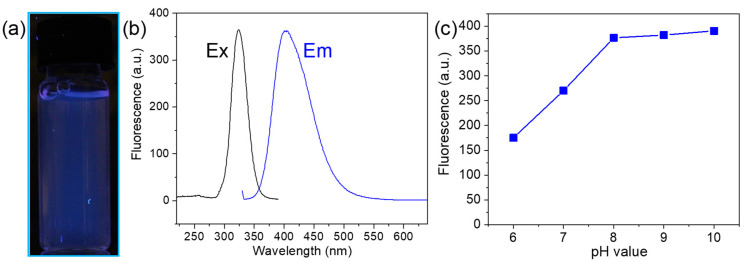
Photoluminescent properties of the Cu@SF NCs. (**a**) Photograph of the Cu@SF NC solution under a 365 nm UV lamp, showing its bright blue emission. (**b**) Fluorescence excitation spectrum (black line) and emission spectra (blue line) of Cu@SF NCs in water. The emission peak is at 400 nm upon excitation at 324 nm. (**c**) Variation in the Cu@SF NC fluorescence intensity (emission at 400 nm) as a function of solution pH, illustrating strong fluorescence in basic conditions and a sharp decrease in acidic conditions.

Importantly, the aqueous Cu@SF NC solution emits a bright blue fluorescence under 365 nm UV illumination (Figure 3a). Under excitation at 324 nm, the emission spectrum shows a peak at ~400 nm (Figure 3b). This blue luminescence arises from the copper nanoclusters themselves, rather than from the SF, since pure SF exhibits only weak fluorescence at 308 nm (Figure S2). We also investigated the effect of pH on the Cu NC fluorescence. The fluorescence intensity remains high and relatively stable at pH 8 and above, but it drops sharply when the pH falls below ~8 (Figure 3c). The diminished emission in more acidic conditions is likely due to protonation of amino acid residues on the SF capping layer, which impedes charge-transfer processes on the Cu NC surface and thus quenches the fluorescence. Based on this behavior, we performed all subsequent sensing experiments in neutral to slightly basic conditions (around pH 8) where the Cu NCs exhibit robust fluorescence.

### 3.3. Diverse Fluorescent Responses of Cu@SF NCs to Different Pollutants

Modern agricultural practices and industrial activities have led to the widespread release of synthetic organic compounds such as pesticides and industrial chemicals into the environment [49,50,51]. This has resulted in increasingly severe pollution problems. It is estimated that millions of people worldwide suffer from pesticide poisoning each year, with over 300,000 fatalities annually [52,53]. Consequently, there is an urgent need for efficient, sensitive, and highly selective detection methods for the rapid identification and quantification of trace pollutants in environmental samples.

**Figure 4:**
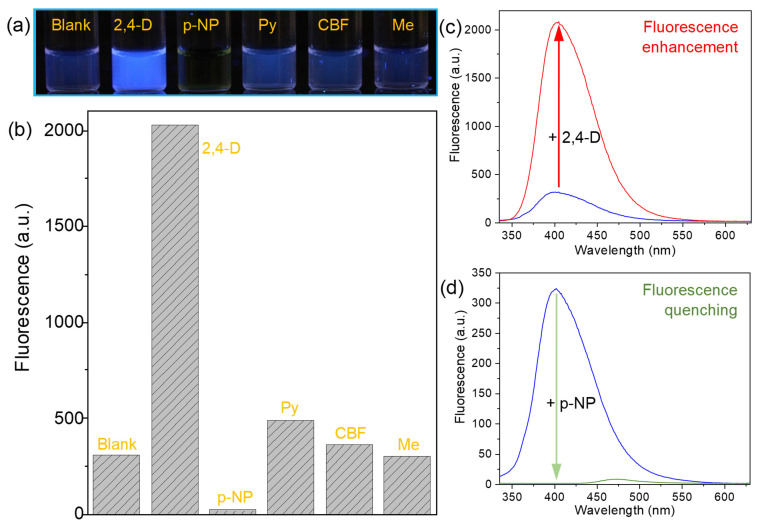
Diverse fluorescence responses of Cu@SF NCs to different pollutants (each at 400 µM, 2 h incubation). (**a**) Fluorescence image of Cu@SF NC samples under a 365 nm UV lamp after adding each pollutant. (**b**) Fluorescence intensities of Cu@SF NCs at 400 nm (excited at 324 nm) for each sample in (**a**). The presence of 2,4-D causes a strong fluorescence enhancement, whereas p-NP causes pronounced quenching. (**c**) Fluorescence emission spectra of Cu@SF NCs before and after the addition of 2,4-D, showing a significant increase in emission intensity (the blue line is the as-synthesized Cu@SF NCs). (**d**) Fluorescence emission spectra of Cu@SF NCs before and after the addition of p-NP, showing nearly complete fluorescence quenching (the blue line is the as-synthesized Cu@SF NCs).

To explore the potential of Cu@SF NCs as a broad sensing platform, we examined their fluorescence response to five representative pollutant molecules: 2,4-D, Py, CBF, p-NP, and Me (the molecular structures are shown in Figure S4). In each case, 400 µM of the analyte was added to the Cu NC solution, and the change in fluorescence was observed before and after addition. The results, summarized in Figure 4, show clearly distinct responses for different analytes. As illustrated in Figure 4a,b, the Cu NC fluorescence at 400 nm remains almost unchanged in the presence of Py, CBF, or Me, indicating a very low sensitivity to those species. In sharp contrast, the addition of 2,4-D produces a dramatic “turn-on” effect—an approximately 6.6-fold increase in the fluorescence intensity (Figure 4c). After considering the solvent effect and the binding of one Cu^2+^ ion with two 2,4-D^−^ ions, we simulated the binding energies of different molecules to Cu^+^ ions (Table S1). The results indicate that 2,4-D exhibits the strongest binding energy (6.71 eV) with Cu^+^ ion. In contrast, Py, CBF, and Me show weaker binding energies of 4.07, 3.91, and 3.62 eV, respectively, consistent with their lower fluorescence responses. Different with the fluorescence enhancement by 2,4-D, the addition of p-NP results in complete quenching of the Cu NC emission at 400 nm (to near-zero intensity; Figure 4d).

These diverse behaviors demonstrate that Cu@SF NCs can differentiate between various pollutants through either fluorescence enhancement or quenching, making them a promising platform for the development of sensitive and selective sensors.

### 3.4. Fluorescence-Enhanced Sensor for Detecting 2,4-D

Developing methods for rapid and ultrasensitive pesticide detection remains challenging due to interference from complex sample matrices and the extremely low concentration of residues that must be detected. 2,4-D is one of the earliest and most widely used synthetic pesticides, largely because of its low cost and effectiveness [54]. However, 2,4-D can persist in the environment (e.g., in soil), and exposure to 2,4-D has been linked to genotoxic, carcinogenic, neurotoxic, and reproductive health effects in humans, as well as developmental abnormalities [55,56]. Therefore, sensitive and selective detection of 2,4-D residues is of great importance for environmental and public health monitoring.

Our results above (Figure 4) indicate that Cu@SF NCs are a promising fluorescent probe for 2,4-D, exhibiting a strong enhancement in fluorescence upon 2,4-D binding. To understand the mechanism of this “turn-on” response, we carried out a series of comparative experiments combined with DFT modeling. The molecular structure of 2,4-D features a phenoxyacetic acid with a carboxyl group, which is the key functional site (Figure S4). In aqueous solution, 2,4-D can dissociate to its anionic form (2,4-D^−^) depending on pH (pKa = 2.73) [57]. Thus, at the pH (~8) of our experiments, 2,4-D will be largely deprotonated. One hypothesis was that protons (H^+^) released from 2,4-D might influence Cu NC fluorescence. However, control tests showed that simply lowering the pH of the Cu NC solution with HCl (from pH 10 to pH 6) actually weakens the NCs’ fluorescence (Figure 3c and Figure S5). This rules out the possibility that any H^+^ generated by 2,4-D is responsible for the fluorescence increase. We also confirmed that ethanol used to introduce 2,4-D has no effect on the Cu NC emission (Figure S5). Additionally, when 400 µM 2,4-D was added to a solution of SF alone (without Cu NCs), it did not enhance the SF’s fluorescence; in fact, a slight decrease in SF fluorescence was observed (Figure S6). Taken together, these observations demonstrate that the fluorescence enhancement is specific to the interaction between 2,4-D and the Cu NCs, rather than a general effect on the SF or solution pH. We attribute the fluorescence turn-on to the effective binding of 2,4-D^−^ anions with the unreacted Cu^2+^ ions, which alters the aggregation state of the nanoclusters.

**Figure 5:**
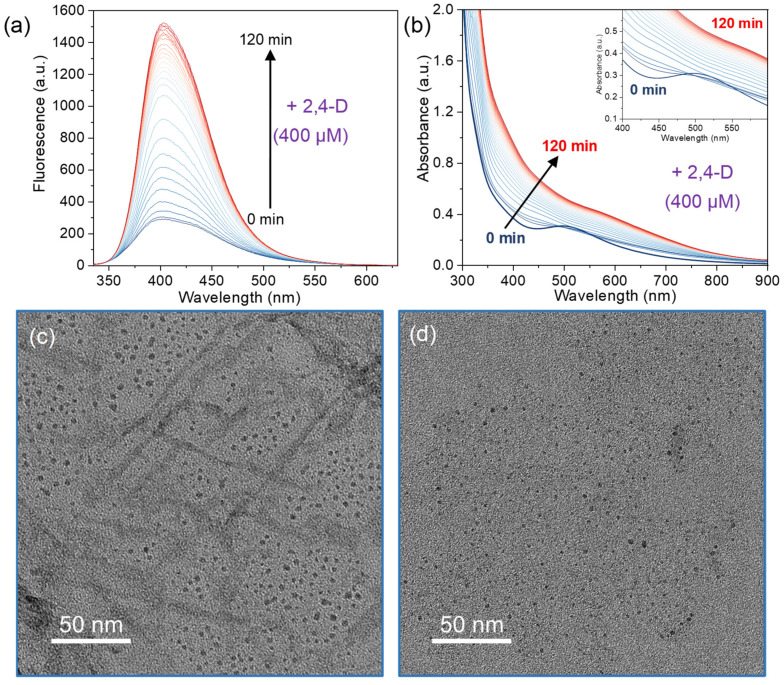
Various characterization of the Cu@SF NCs before and after adding 400 µM 2,4-D. (**a**) Time-resolved fluorescence spectra of the Cu@SF NC solution after adding 2,4-D, showing the gradual increase in emission (λ_em_ = 400 nm) over time. (**b**) Time-resolved UV–vis absorption spectra of the Cu@SF NC solution after adding 2,4-D. The initial Cu NCs absorption peak at 508 nm disappears upon 2,4-D addition and then the absorption band gradually intensifies over time. (**c**) TEM image of as-synthesized Cu@SF NCs with an average size of ~5 nm; (**d**) TEM image of 2,4-D-adsorbed Cu@SF NCs with an average size of 2.5 nm.

To gain further insight, we examined the fluorescence enhancement kinetics at different 2,4-D concentrations. As shown in Figure 5a and Figure S7, the Cu NC fluorescence gradually increases over time after adding 2,4-D, eventually reaching a plateau. This time-dependent behavior was similar for 2,4-D concentrations of 100 µM, 200 µM, and 400 µM. In each case, the fluorescence intensity approached a steady maximum after about 120 min of incubation (Figure S8). Higher concentrations of 2,4-D produced a larger overall fluorescence increase (higher plateau value). At an even higher 2,4-D concentration (800 µM), the fluorescence reached its maximum more quickly—within approximately 60 min (Figure S9). It was found that reducing the concentration of NCs can accelerate the sensing kinetics and shorten the incubation time. Additionally, we investigated the optimal pH environment for detecting 2,4-D. As shown in Figure S10, Cu NCs exhibited the highest sensing sensitivity at pH 8. Moreover, Cu NCs exhibit highly stable fluorescence in both water and PBS buffer at pH 8. The fluorescence responses to 2,4-D are essentially identical in both media (Figure S11).

Additional evidence for the disaggregation mechanism was obtained from UV–vis and TEM analyses. Immediately after adding 2,4-D to the Cu NC solution, the characteristic 508 nm absorbance peak of the Cu NCs was greatly diminished, indicating disruption of the original clusters. With continued incubation, the overall absorbance of the solution gradually increased (Figure 5b). Consistent with this, the decrease in Cu NCs in size was confirmed by TEM characterization, where the as-synthesized Cu@SF NCs exhibited a size of approximately 5 nm (Figure 5c), and the introduction of 2,4-D resulted in monodisperse NCs with a size of approximately 2.5 nm (Figure 5d). The reduction in metal particles’ size accompanied by fluorescent enhancement are usually elucidated as the disaggregation of aggregated NCs or/and the etching of ligands to nanoparticles. Notedly, the etching process from large nanoparticles into small NCs will lead to a clear blue-shift of fluorescent peak with incubation time. Owing that there is no change in emission wavelength of Cu NCs after adding 2,4-D (Figure 5a), these results suggest that the Cu NCs, which initially presented as small aggregates, become more finely dispersed in the presence of 2,4-D.

Based on the above observations, we propose the mechanism illustrated in Figure S12. The SF template is composed mostly of small amino acids (Gly, Ala, Ser) and contains only a few residues with aromatic or strongly coordinating side chains (e.g., Tyr). At the used ratio of precursors (i.e., 2 mM Cu^2+^ with 10 mg/mL SF), the unreacted Cu^2+^ ions are existed in the Cu@SF NCs solution. This leads to slight aggregation of the as-synthesized Cu NCs into larger assemblies, which in turn results in relatively weak fluorescence (likely due to interactions or energy transfer between adjacent Cu cores in the aggregate). Because Gly’s side chain is just a hydrogen (non-electron-donating), the SF molecules only exhibit a weak binding interaction with Cu^2+^ ions. When these aggregated Cu NCs encounter 2,4-D in solution (at pH 8), the 2,4-D molecules deprotonate and the resulting anions bind with Cu^2+^ ions more strongly [58]. The strong binding of 2,4-D^−^ facilitates the release of Cu^2+^ from the aggregated Cu NCs and causes them to redisperse into smaller, isolated NCs. Consequently, the fluorescence is restored and greatly enhanced [59]. DFT calculations support this interpretation (Table S1): a 2,4-D^−^ anion can form a bidentate coordination with a Cu^+^ through its carboxylate oxygen and ether oxygen (the binding of one Cu^2+^ ion with two 2,4-D^−^ ions, Figure S12a), and this mode of binding has a high calculated energy (E_bind_ = 6.71 eV). Such strong binding effectively “locks” the 2,4-D^−^ with Cu^2+^ ions, indirectly helping to stabilize the cluster and prevent it from aggregating [60]. The net result is a significant increase in fluorescence intensity. To validate this mechanism, we further evaluated the fluorescent response of MCPA and dicamba on Cu NCs, which share a similar structure to 2,4-D. As shown in Figure S13, all three molecules with carboxylate anions induced fluorescence enhancement of Cu NCs, with 2,4-D exhibiting the most pronounced enhancement effect.

**Figure 6:**
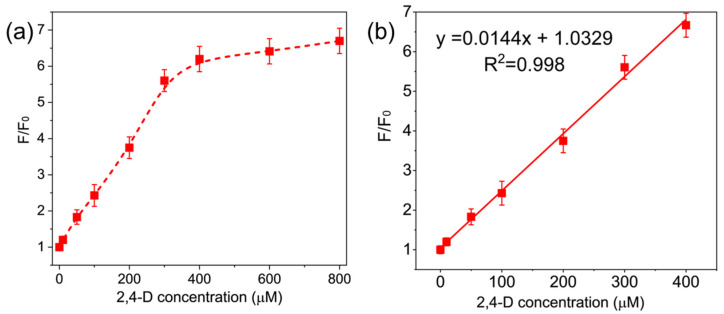
(**a**) Fluorescence enhancement of Cu@SF NCs (plotted as F/F_0_) as a function of 2,4-D concentration after adding different concentrations of 2,4-D. (**b**) Calibration curve for 2,4-D detection: the ratio F/F_0_ after 120 min incubation is plotted against 2,4-D concentration (0–400 µM). A linear fit to the data yields F/F_0_ = 0.0144 × [2,4-D] + 1.0329 (R^2^ = 0.998). The detection limit for 2,4-D is 0.65 µM (3σ criterion).

With the mechanism of the fluorescence-on response understood, we optimized the Cu@SF NC system as a sensor for 2,4-D. As noted earlier, ethanol (used to deliver 2,4-D) does not interfere with the fluorescence (Figure S5). We therefore constructed a calibration by adding increasing concentrations of 2,4-D (dissolved in ethanol) to the Cu NC solution and measuring the fluorescence after 120 min of incubation. The fluorescence intensity (F) at 400 nm, normalized to the initial intensity without analyte (F_0_), is plotted as a function of 2,4-D concentration in Figure 6a. The F/F_0_ value increases systematically with rising 2,4-D concentration, showing a good linear relationship in the range from 0 to 400 µM (Figure 6b). The linear fit is F/F_0_ = 0.0144 × [2,4-D] + 1.0329, with a correlation coefficient R^2^ = 0.998. The limit of detection (LOD), calculated as 3σ/S (where σ is the standard deviation of blank measurements and S is the slope of the calibration line), is 0.65 µM. These analytical figures of merit demonstrate that the Cu@SF NCs provide a simple yet highly sensitive method for detecting 2,4-D. The probe’s strong fluorescence response and high selectivity suggest it could serve as a reliable platform for monitoring 2,4-D residues in environmental samples.

### 3.5. Fluorescence-Quenched Sensor for Detecting p-NP Molecules

Industrial wastewater discharge is a major source of water pollution, often containing toxic phenolic compounds. p-NP is a priority pollutant frequently found in the effluents of chemical manufacturing and is of great concern in environmental monitoring [61]. We found that p-NP causes a pronounced quenching of the Cu@SF NC fluorescence (Figure 4d), making this system potentially useful for detecting p-NP via a “fluorescence-off” mechanism.

**Figure 7:**
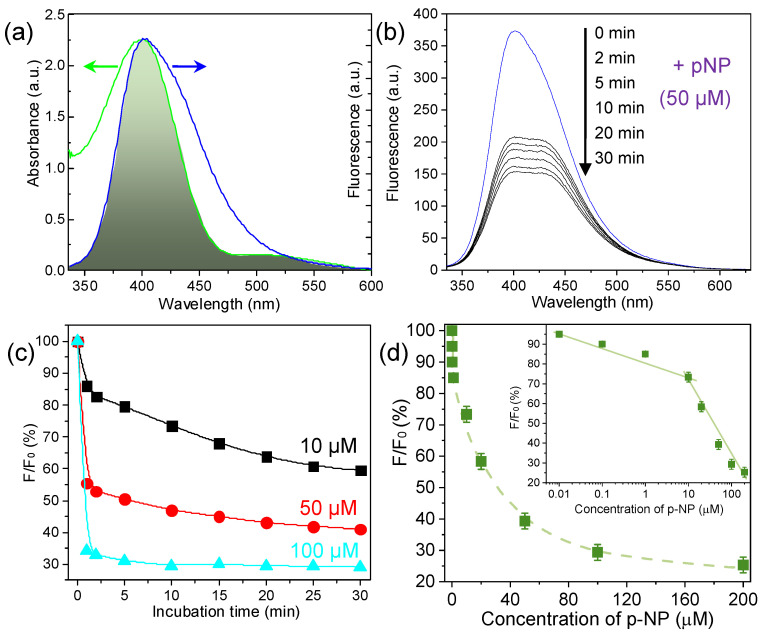
Fluorescence-quenching detection of p-NP using Cu@SF NCs. (**a**) Spectral overlap between the Cu@SF fluorescence emission (donor, blue curve and arrow) and the p-NP absorption spectrum (acceptor, green curve and arrow). The significant overlap (350–450 nm) suggests a FRET-based quenching mechanism. (**b**) Time-dependent decrease in Cu@SF fluorescence after adding 50 µM p-NP, reaching a steady state by ~30 min. (**c**) Fluorescence quenching kinetics at different p-NP concentrations (10, 50, 100 µM). Higher p-NP levels cause faster and greater quenching. (**d**) Calibration plot for p-NP detection: F/F_0_ is plotted against p-NP concentration. The inset shows linear relationships (logarithmic scale) in two concentration ranges: 0.1–10 µM and 10–200 µM. The Cu@SF NC sensor achieves an LOD of 1.35 nM for p-NP.

To clarify how p-NP quenches the Cu NC fluorescence, we examined the spectral overlap between the Cu NC emission and the p-NP absorption. As shown in Figure 7a, p-NP has a broad UV–vis absorption band spanning roughly 350–450 nm, which overlaps significantly with the Cu NCs’ fluorescence emission spectrum (centered at 400 nm). Such a strong overlap is a hallmark of the fluorescence resonance energy transfer (FRET) mechanism of quenching [62,63]. In a FRET process, the fluorescent donor’s emission is non-radiatively transferred to an acceptor (quencher) if the donor’s emission spectrum overlaps the acceptor’s absorption spectrum, and the efficiency of energy transfer increases with the degree of spectral overlap [64]. In our case, the Cu NCs act as the energy donor and p-NP as the acceptor; the substantial overlap between the Cu NC emission and p-NP absorption fulfills the key requirement for FRET. We therefore attribute the fluorescence quenching primarily to a FRET mechanism from the excited Cu NCs to the p-NP molecules, which absorb the energy.

We next studied the quenching behavior as a function of p-NP concentration and time. The Cu NC fluorescence decreased for all tested p-NP concentrations, and we determined that a 30 min incubation is sufficient for the quenching to reach equilibrium (Figure 7b and Figure S14). We monitored the fluorescence intensity over time at several p-NP concentrations (e.g., 10 µM, 50 µM, 100 µM; see Figure 7c). The quenching occurred faster and to a greater extent at higher p-NP concentrations: at 100 µM p-NP, about 70% of the Cu NC fluorescence was quenched within 30 min, whereas at 10 µM p-NP, roughly 40% quenching was observed in the same period. This concentration-dependent quenching rate suggests a dynamic collisional or associative interaction between p-NP and the Cu NCs that is more efficient at higher p-NP levels, enabling quantitative analysis of p-NP via its quenching effect.

Finally, we constructed a calibration curve for p-NP detection. Figure 7d shows the relationship between the normalized fluorescence (F/F_0_) and the p-NP concentration. The fluorescence ratio decreases monotonically as p-NP concentration increases. The inset of Figure 7d highlights two linear response ranges on a logarithmic concentration scale: from 0.1 µM to 10 µM and from 10 µM to 200 µM. In the low concentration range (0.1–10 µM), the data fit a linear model of F/F_0_ = −7.1 × log[p-NP] + 82.2, with R^2^ = 0.955. In the higher range (10–200 µM), the linear fit is F/F_0_ = −38.1 × log[p-NP] + 108.1, with R^2^ = 0.965. The LOD for p-NP (using 3σ/S) was calculated to be 1.35 nM. Additionally, under pH 8 condition, Cu NCs can effectively detect p-NP concentrations in tap water, lake water and rainwater, with a relative error not exceeding 2%. These results demonstrate that the Cu@SF NC probe can serve as a highly sensitive sensor for p-NP over a broad range of concentrations. The combination of a strong quenching response, low detection limit, and two-range linearity makes it suitable for detecting p-NP in environmental or possibly biological samples.

## 4. Conclusions

In summary, we developed a simple, green strategy to prepare fluorescent Cu nanoclusters using regenerated SF as both a reducing agent and a stabilizing template, which are at a slightly aggregated state via the unreacted Cu^2+^ ions. The SF extraction method (using a 50% CaCl_2_ solution) yielded highly soluble fibroin, which enabled the one-pot synthesis of water-dispersible Cu@SF NCs under mild alkaline conditions. The resulting Cu NCs exhibit bright blue emission with a peak at 400 nm (under 324 nm excitation), attributable to effective coordination between SF and copper ions and the successful reduction of Cu^2+^ to Cu^0^/Cu^+^. Notably, the slightly aggregated Cu@SF NCs show distinct fluorescence responses toward different analytes, allowing the construction of a multifunctional sensing platform. In the presence of the pesticide 2,4-D, the deprotonated 2,4-D^−^ anions bind with Cu^2+^ ions more strongly and release them from Cu NCs, disrupting NC aggregation and turning “on” the fluorescence. The Cu NC sensor for 2,4-D provides a broad linear detection range (0–400 µM) and a low LOD (0.65 µM), demonstrating excellent performance for trace 2,4-D detection. In contrast, the pollutant p-NP strongly quenches the Cu NC fluorescence via a FRET mechanism, effectively turning the sensor “off”. This Cu NC probe achieves an LOD of 1.35 nM for p-NP and shows good linearity across two concentration ranges (0.1–10 µM and 10–200 µM), underscoring its sensitivity for p-NP monitoring. Other tested compounds (Py, CBF, Me) produced negligible fluorescence changes, confirming the high selectivity of the sensing system.

Through systematic experimental characterization (UV–vis, photoluminescence, XPS, TEM) and mechanism studies (including DFT calculations), we identified two distinct sensing mechanisms: 2,4-D enhances Cu NCs’ fluorescence by disaggregating the clusters, whereas p-NP quenches the fluorescence via energy transfer. These insights deepen our understanding of how protein templates influence the structure and optical behavior of metal nanoclusters, and they provide useful guidance for designing stimuli-responsive fluorescent nanomaterials. Furthermore, this work expands the application scope of non-precious metal nanoclusters in the field of chemical sensing. By rationally selecting appropriate template molecules (such as fibrous proteins like SF), one can optimize the optical properties and improve the sensing performance of metal NCs, thereby providing a practical approach for environmental pollutant detection and monitoring.

## Data Availability

Data supporting the findings of this study are contained within the article and its Supplementary Materials.

## Supplementary Materials

The following supporting information can be downloaded at: https://www.mdpi.com/article/10.3390/s26030784/s1, Figure S1: Sodium dodecyl sulfate polyacrylamide gel electrophoresis (SDS-PAGE) analysis of the silk fibroin solution (molecular weight distribution); Figure S2: Spectroscopic properties of the silk fibroin solution; Figure S3: Optical images of Cu^2+^ solution, SF solution, a Cu^2+^ + SF mixture, and the resulting Cu@SF NC solution; Figure S4: Molecular structures of the pollutant analytes; Table S1: The calculated binding energies between different organic molecules and Cu^+^ ions; Figure S5: Influence of various species on the fluorescence of Cu@SF NCs; Figure S6: Fluorescence spectra of silk fibroin after adding 400 µM 2,4-D; Figure S7: Time-dependent fluorescence spectra of the Cu@SF NC solution after adding 100 µM and 200 µM 2,4-D; Figure S8: Temporal evolution of Cu@SF NC fluorescence enhancement for different 2,4-D concentrations; Figure S9: Time-dependent fluorescence spectra and fluorescence enhancement of Cu@SF NCs after adding 800 µM 2,4-D (showing saturation by 60 min); Figure S10: Effect of pH value on fluorescence enhancement after adding 200 µM 2,4-D into the Cu NCs solution; Figure S11: Comparison of Cu NCs’ detection performance for 2,4-D in H_2_O and PBS buffer solutions; Figure S12: TEM image of slightly aggregated Cu@SF NCs and Cu@SF NCs after adding 2,4-D; Figure S13: Fluorescence response of 2,4-D structural analogues toward Cu NCs; Figure S14: Time-dependent fluorescence spectra of the Cu@SF NC solution after adding 10 µM and 100 µM p-NP.
