# Supplementary material for: Silk Fibroin-Templated Copper Nanoclusters: Responsive Fluorescent Probes Exhibiting 2,4-Dichlorophenoxyacetic Acid-Enhanced Emission and p-Nitrophenol-Induced Quenching
Source: Sensors (Basel). 2026 Jan 24;26(3):784. doi: 10.3390/s26030784 (PMC12899482; doi:10.3390/s26030784)
Supplement: Supplementary file 1 [file sensors-26-00784-s001.zip › sensors-4042407-supplementary.pdf]

Supporting Information

Silk Fibroin-Templated Copper Nanoclusters: Responsive Fluorescent Probes Exhibiting 2,4-Dichlorophenoxyacetic Acid-Enhanced Emission and p-Nitrophenol-Induced Quenching

Neng Qin[1,†], Qian Wang[1,†], Jingwen Tao[1], Guijian Guan[1,2*] and Ming-Yong Han

[1]Institute of Molecular Plus, Tianjin University, Tianjin, 300072, China; qinneng_1@tju.edu.cn (N.Q.); 2024239009@tju.edu.cn (Q.W.); tjw_123_wh@tju.edu.cn (J.T.); han_mingyong@tju.edu.cn (M.-Y.H.)

[2]State Key Laboratory of Advanced Papermaking and Paper-based Materials, South China University of Technology, Guangzhou, 510640, China.

*Corresponding authors

E-mail: guijianguan@tju.edu.cn (G. Guan).

[†]These authors contributed equally to this work.

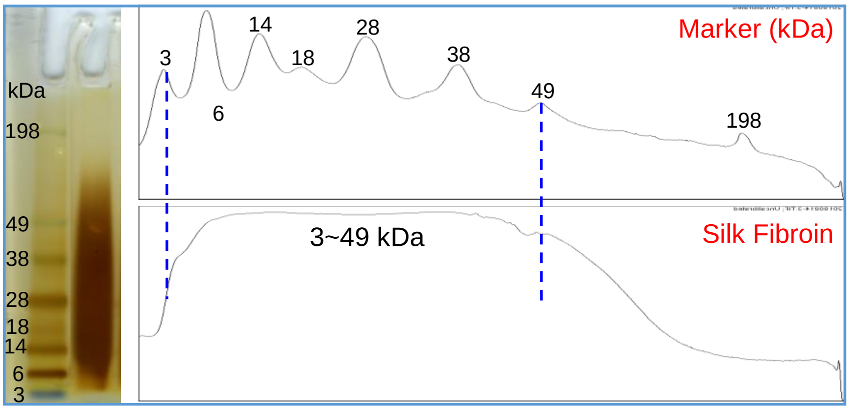

Figure S1. SDS-PAGE (sodium dodecyl sulfate polyacrylamide gel electrophoresis) analysis of the silk fibroin solution, illustrating its molecular weight distribution.

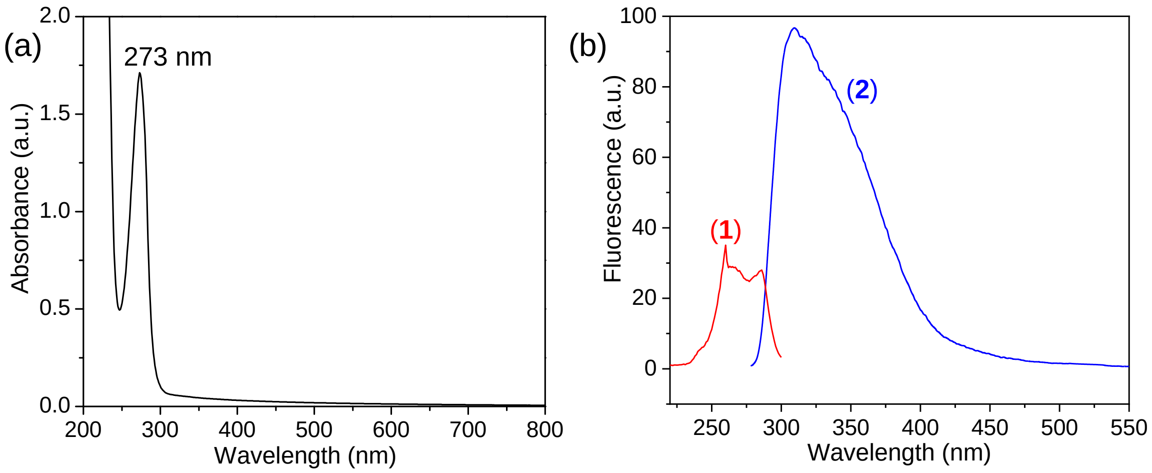

Figure S2. Spectroscopic properties of a silk fibroin solution (2 mg/mL in distilled water): (a) UV-vis absorption spectrum; (b) fluorescence spectra, including (**1**) an excitation spectrum recorded at an emission wavelength of 308 nm and (**2**) an emission spectrum under 260 nm excitation.

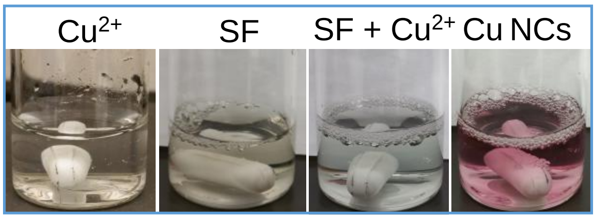

Figure S3. Optical images of four solutions: a 2 mM Cu^{2+} solution; a 10 mg/mL SF solution; a fresh mixture of 2 mM Cu^{2+} and 10 mg/mL SF (i.e., before reaction); and the resulting solution of as-synthesized Cu@SF NCs (i.e., 2 mM Cu^{2+} and 10 mg/mL SF are incubated at 40°C and pH 11 for 4 h).

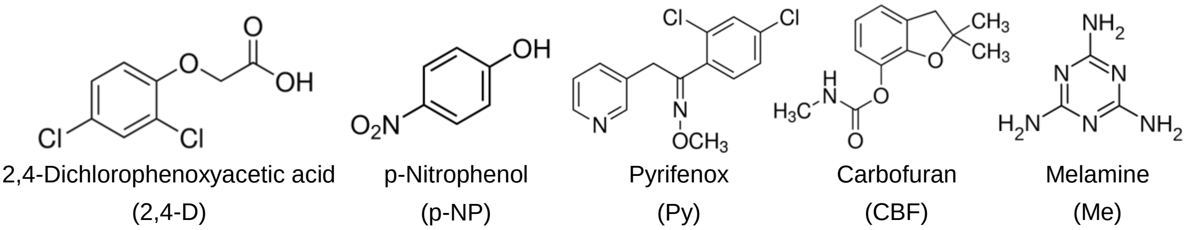

Figure S4. Molecular structures of various pollutant compounds used as model analytes to investigative the diverse fluorescence responses of Cu@SF NCs.

Table S1. The calculated binding energies between different organic molecules and Cu^{+} ions, which are used to reveal the sensing selectivity of Cu NCs.

**Pairs**	**Cu^{+}-2,4-D^{-}**	**Cu^{+}-Py**	**Cu^{+}-CBF**	**Cu^{+}-Me**
**Model**	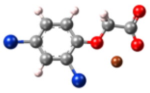	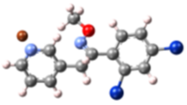	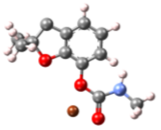	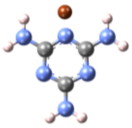
**Binding energy (eV)**	**6.71**	**4.07**	**3.91**	**3.62**

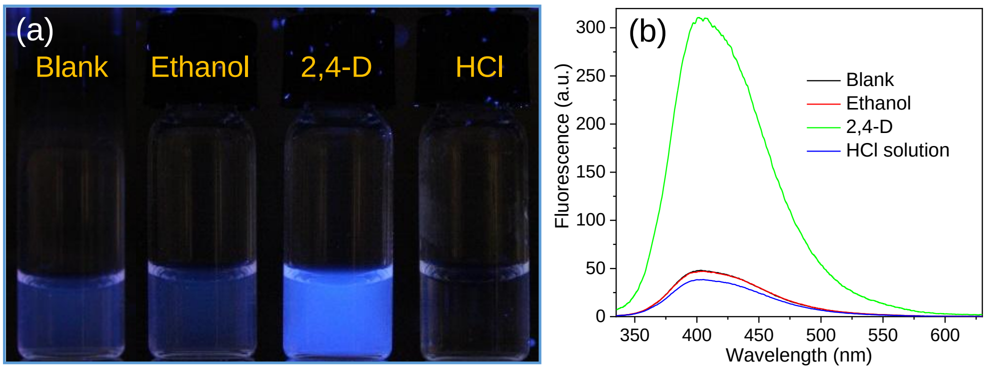

Figure S5. Influence of various added species on the fluorescence of Cu@SF NCs. (a) Fluorescence image of Cu@SF NC solution under a UV lamp after adding different species. (b) Corresponding fluorescence emission spectra. These comparisons highlight the critical role of 2,4-D in enhancing the fluorescence of Cu@SF NCs.

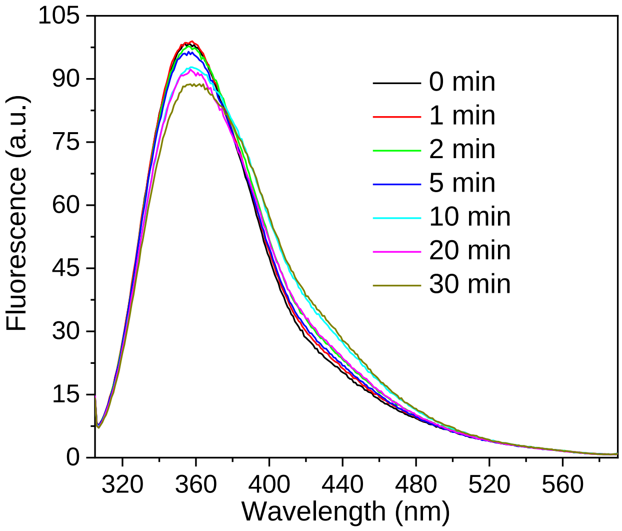

Figure S6. Florescence spectra of a SF solution (pH 11) after the addition of 400 µM 2,4-D (excitation at 298 nm). The results show that 2,4-D does not enhance the native fluorescence of silk fibroin.

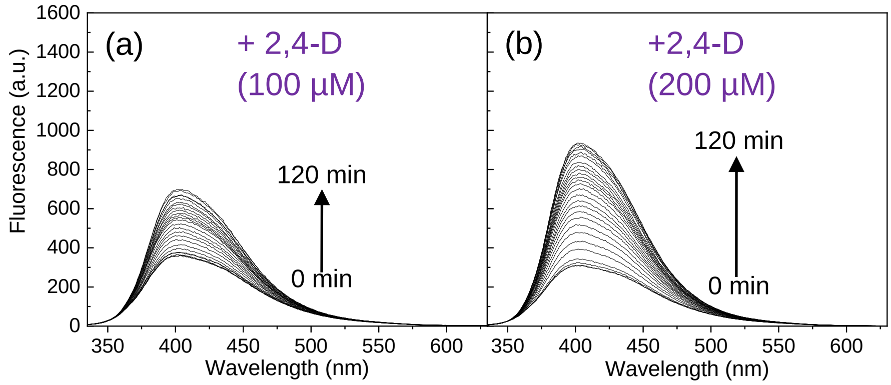

Figure S7. Time-dependent fluorescence spectra of the Cu@SF NCs solution after the addition of 2,4-D at concentrations of (a) 100 μM and (b) 200 μM.

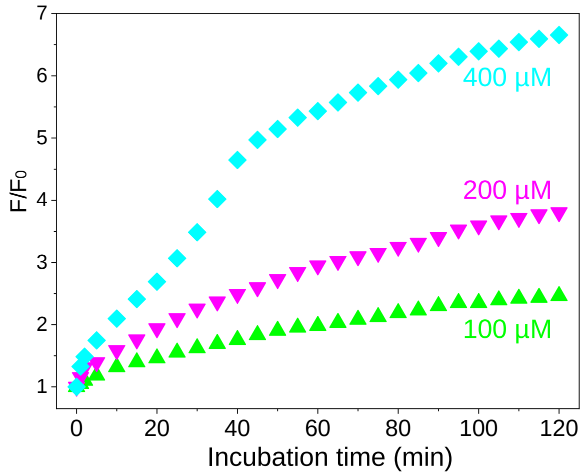

Figure S8. Temporal evolution of the fluorescence enhancement of Cu@SF NCs after adding different concentrations of 2,4-D.

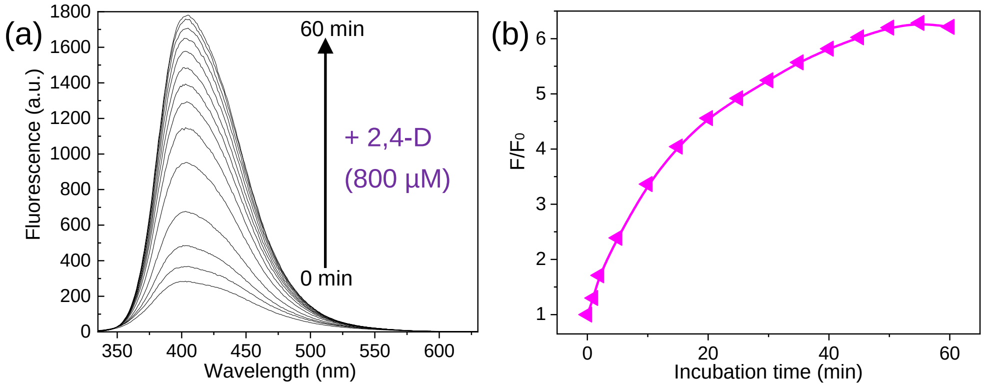

Figure S9. Fluorescence response of Cu@SF NCs upon addition of 800 μM 2,4-D. (a) Fluorescence spectra recorded at different time after adding 2,4-D. (b) Corresponding plot of fluorescence intensity vs. time. Temporal evolution of fluorescence enhancement after adding 800 μM 2,4-D into the Cu@SF NCs solution. The fluorescence reaches its maximum after approximately 60 min of incubation.

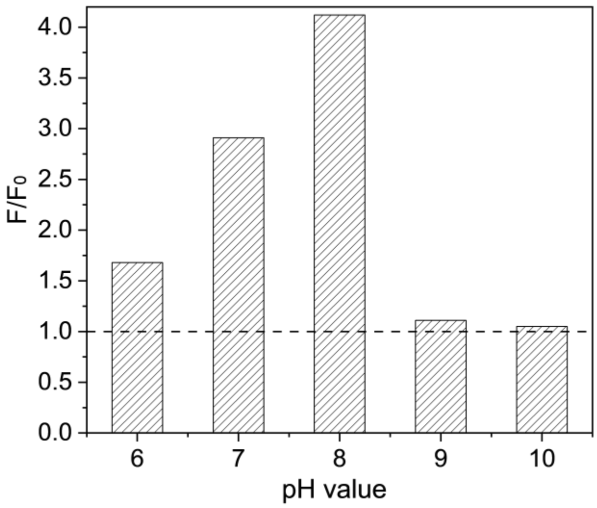

Figure S10. Effect of pH on fluorescence enhancement after adding 400 μM 2,4-D into the Cu NCs solution for 30 min.

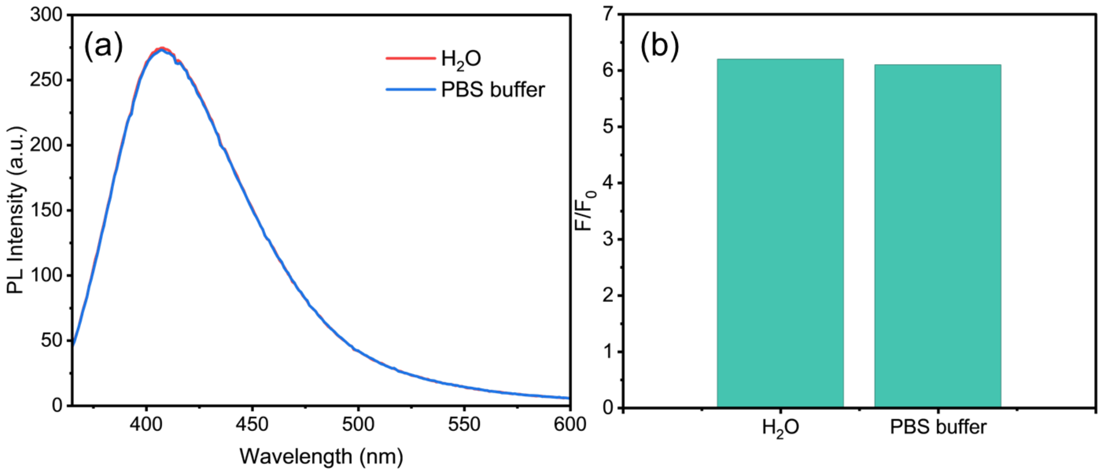

Figure S11. (a) Emission spectra of Cu NCs diluted into the pH 8 solution and PBS buffer (pH=8); (b) Fluorescence response of Cu NCs to 400 μM 2,4-D in the pH 8 solution and PBS buffer (pH=8).

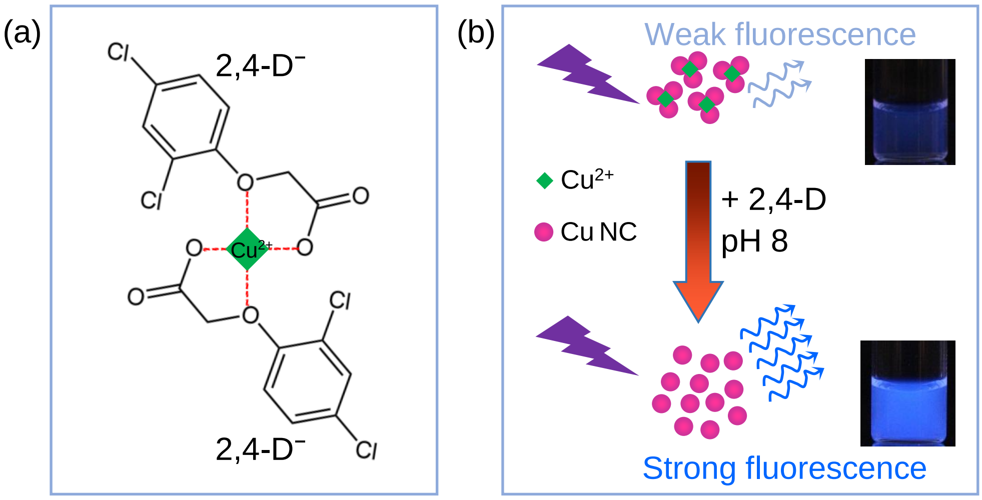

Figure S12. Schematic illustration of the proposed fluorescence enhancement mechanism. (a) The coordinated structure of one Cu^{2+} cation with two 2,4-D⁻ anions [S1]. (b) The as-synthesized Cu@SF NCs initially form small aggregates via the unreacted Cu^{2+} ions, which emit a weak blue fluorescence [S2,S3]; Upon introduction of 2,4-D at pH 8, the 2,4-D⁻ anions take the Cu^{2+} cations away to produce well-dispersed Cu NCs, accompanied by an enhanced fluorescence [S3].

[S1] Alhamami, M.A.M.; Mohammed, A.Y.A.; Algethami, J. S.; Al-Saidi, H. M.; Khan, S.; Alharthi, S.S. Highly sensitive and selective Schiff base chemosensor for Cu^{2+} and 2,4-D detection: A promising analytical approach. *Microchem. J.* **2024**, *197*, 109817.

[S2] Yang, J.; Song, N.; Lv, X.; Jia, Q. UV-light-induced synthesis of PEI-CuNCs based on Cu^{2+}-quenched fluorescence turn-on assay for sensitive detection of biothiols, acetylcholinesterase activity and inhibitor. *Sens. Actuators B Chem.* **2018**, *259*, 226-232.

[S3] Wang, W.; Zhan, L.; Du, Y.Q.; Leng, F.; Huang, C.Z. A new spectrofluorometric method for pyrophosphate assay based on the fluorescence enhancement of trypsin-stabilized copper clusters. *Anal. Methods* **2015**, *7*, 638.

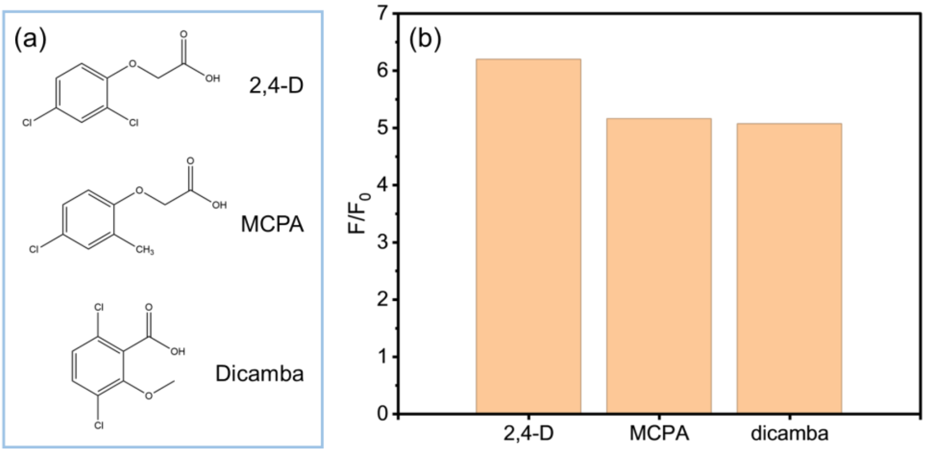

Figure S13. (a) Molecular structure of 2,4-D, MPCA and dicamba. (b) Fluorescence responses of Cu NCs under exposure to 400 µM 2,4-D, MCPA, and dicamba, respectively.

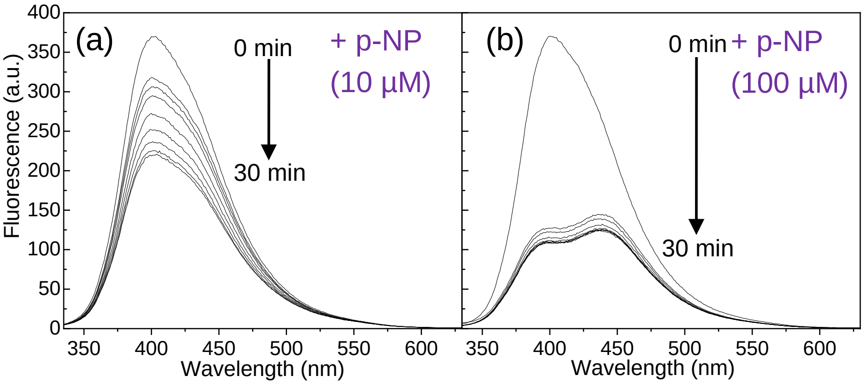

Figure S14. Time-dependent fluorescence spectra of the Cu@SF NCs solution after the addition of p-NP at two concentrations: (a) 10 µM and (b) 100 µM.
